# Genetic evaluation of small ruminant lentivirus susceptibility in Valais blacknose sheep

**DOI:** 10.1111/age.13108

**Published:** 2021-06-24

**Authors:** Anna Letko, Charis Bützberger, Nathalie Hirter, Julia M. Paris, Carlos Abril, Cord Drögemüller

**Affiliations:** ^1^ Institute of Genetics, Vetsuisse Faculty University of Bern Bremgartenstrasse 109a Bern 3001 Switzerland; ^2^ Institute of Virology and Immunology, Vetsuisse Faculty University of Bern Länggassstrasse 122 Bern 3001 Switzerland

## Background

Maedi‐Visna is a complex production‐limiting ovine lentiviral disease caused by small ruminant lentivirus (SRLV) infection widespread throughout the world characterised by long immunological and clinical latencies and chronic progressive inflammatory pathology.^1^ Previously, an SNP in the ovine *TMEM154* gene (Oar_rambouillet_v1.0: chr 17:g.5776842G>A) as well as a short indel variant in the promoter region of ovine *CCR5* (Oar_rambouillet_v1.0: chr 19:g.54980459_54980460ins) were reported as markers associated with resistance/susceptibility to SRLV in several sheep breeds globally (OMIA 001694‐9940).^2^ ^,^ ^3^ Multiple other genomic regions associated with susceptibility to or control of infection were also reported indicating genetic complexity.^4^ In Switzerland the Valais blacknose sheep (VBS) breed was found to have the highest prevalence of Maedi‐Visna among Swiss sheep breeds.^5^

## Own analysis

We collected pairs of SRLV‐infected and non‐infected individuals from this native Swiss sheep breed (Table S1). All initially positive tested sheep for SRLV were determined seropositive by two commercial ELISA tests: (1) Checkit CAEV/MVV®ELISA (IDEXX Laboratories, Liebefeld, Switzerland), a whole‐virus antigen‐based indirect ELISA; and (2) Small Ruminant Lentivirus Antibody Test Kit (VMRD, Pullman, WA, USA), a genotype B gp135 competitive ELISA. Positive samples were confirmed subsequently by western blotting focusing on the detection of the viral capsid (p25), matrix (p18), and nucleocapsid (p15) proteins^6^ in the Swiss national reference laboratory for lentiviruses in small ruminants. The controls were ELISA‐based negative tested only. The average age of the confirmed SRLV‐positively tested sheep designated as cases was 5 years ranging from 2 to 9 years, whereas the SRLV‐negative flock mates selected as controls were on average 4.6 years old (ranging from 1 to 11 years; Table S1). Subsequently all 67 animals were genotyped for the previously described *TMEM154* ^2^ and *CCR5* ^3^ variants by direct Sanger sequencing using the ABI 3730 DNA Analyzer (Thermo Fisher Scientific, Reinach, Switzerland). Therefore, genomic DNA was extracted from EDTA‐stabilized blood samples using the Maxwell RSC instrument (Promega, Dübendorf, Switzerland). Interestingly, no homozygous wild type genotypes were observed for the *TMEM154* marker, while 29% of cases and 3% of controls were heterozygous. For the *CCR5* marker, 65% of cases and 73% of controls had the homozygous wild type genotype, while 26% of cases and 12% of controls were heterozygous (Table 1). We observed neither a significant association nor a trend while comparing the genotypes with the SRLV infection status.

**Table 1:** Genotypes of the 67 Valais blacknose sheep in the two previously reported markers^2^ ^,^ ^3^

SRLV serostatus	*TMEM154*			*CCR5*		
	GG	GA	AA	wt/wt	wt/del	del/del
Positive (n=34)	0	10	24	22	9	3
Negative (n=33)	0	1	32	24	4	5

Subsequently all 67 animals (34 cases and 33 controls) were genotyped using the Illumina ovine HD BeadChip.^7^ After quality control (call rate >90%, minor allele frequency >0.05), 67 animals and 416,454 SNPs were retained. Genotyping data can be retrieved at https://osf.io/b35ud/ (https://doi.org/10.17605/OSF.IO/FSRPW). Using gemma v0.98,^8^ a genome‐wide association study (GWAS) was conducted to look for additional genome regions associated with susceptibility to MV in VBS. To show the genetic distances among the studied animals, a relatedness matrix was generated (Fig. S1). GWAS revealed the best‐associated SNP (*P*‐value = 8.2 × 10^−5^) on chromosome 9 at 69 843 937 bp (Fig. S1) although it did not reach the Bonferroni corrected genome‐wide significance level (‐log(*P*‐value) = 6.9). Eight out of the 10 best‐associated SNPs map to this genome region from 62 to 72 Mb at chromosome 9 (Table S2). Based on the NCBI *Ovis aries* annotation release 103, a total of 42 genes and loci are annotated in that 10‐Mb region of the Oar_rambouillet_v1.0 assembly (Table S3).

## Conclusions

No association between genotypes in the *TMEM154* and *CCR5* and SRLV susceptibility in the studied local Swiss breed VBS could be determined showing that these two previously reported genetic markers do not affect individual susceptibility to infection in this particular breed. Despite that our study was performed on a limited number of individuals, similar to a recent GWAS performed comparing 21 serologically positive with 27 negative tested goats of an Italian native breed,^9^ it also suggests further genetic complexity underlying the resistance/susceptibility to SRLV in sheep.

## Supporting information


**Figure S1**. (a) Multidimensional scaling plots of genetic relationships among the 67 VBS in the first three coordinates. (b) Manhattan plot of ‐log(*P*‐values) for the genome wide association study.

**Table S1**. Features of the 67 animals used for this study.

**Table S2**. GWAS results.

**Table S3**. List of annotated genes and loci in the associated genome region.

## References

[age13108-bib-0001] Gomez‐Lucia E. *et al*. (2018) Vet Med Res Reports 9, 11–21. 10.2147/VMRR.S136705 PMC6042483 30050863

[age13108-bib-0002] Heaton M.P. *et al*. (2012) PLoS Genetics 8, e1002467. 22291605 10.1371/journal.pgen.1002467 PMC3266874

[age13108-bib-0003] White S.N. *et al*. (2009) Animal Genetics 40, 583–9. 19397512 10.1111/j.1365-2052.2009.01882.x

[age13108-bib-0004] White S.N. *et al*. (2012) PLoS One 7, e47829. 23082221 10.1371/journal.pone.0047829 PMC3474742

[age13108-bib-0005] Schaller P. *et al*. (2000) Schweiz Arch Tierheilkunde 142, 145–53. 10804839

[age13108-bib-0006] Zanoni R. *et al*. (1989) J Clin Microbiol 27, 580–2. 2541170 10.1128/jcm.27.3.580-582.1989 PMC267367

[age13108-bib-0007] Kijas J.W. *et al*. (2014) Animal Genetics 45, 754–7. 25040320 10.1111/age.12197

[age13108-bib-0008] Zhou X. & Stephens M. (2012) Nat Genetics 44, 821–4. 22706312 10.1038/ng.2310 PMC3386377

[age13108-bib-0009] Cecchi F. *et al*. (2019) Trop Animal Health Prod 51, 729–33. 10.1007/s11250-018-1728-y 30350159

