# Supplementary figures and images for: Genetic evaluation of small ruminant lentivirus susceptibility in Valais blacknose sheep
Source: Anim Genet. 2021 Jun 24;52(5):781–2. doi: 10.1111/age.13108 (PMC8518827; doi:10.1111/age.13108)

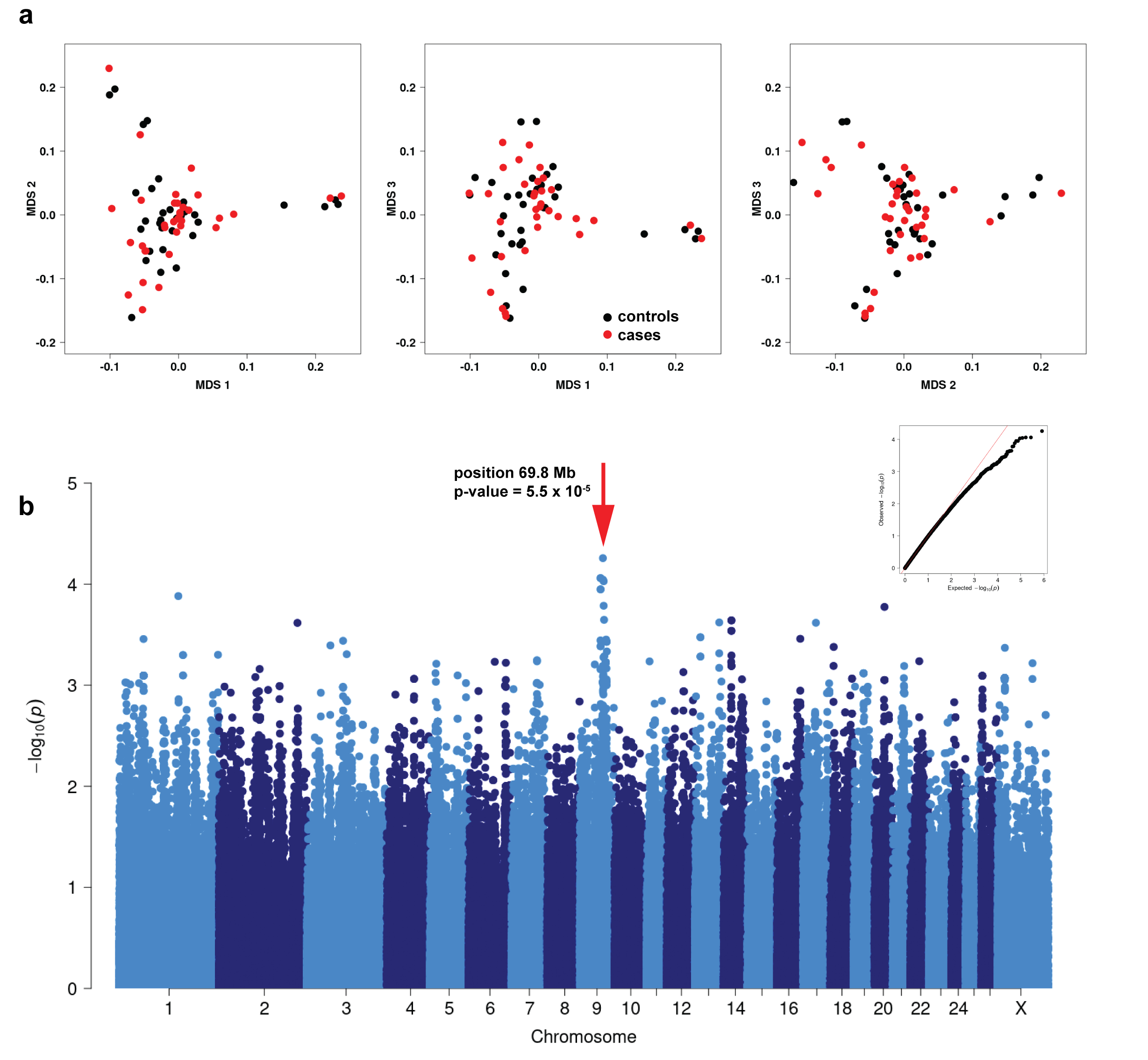

Supplement: Supplementary file 1 — Figure S1. (a) Multidimensional scaling plots of genetic relationships among the 67 VBS in the first three coordinates. (b) Manhattan plot of ‐log(P‐values) for the genome wide association study. [file AGE-52-781-s002.tif]
